# Histone Deacetylase 3 Aggravates Type 1 Diabetes Mellitus by Inhibiting Lymphocyte Apoptosis Through the *microRNA-296-5p/Bcl-xl* Axis

**DOI:** 10.3389/fgene.2020.536854

**Published:** 2020-11-02

**Authors:** Qibo Hu, Guanghua Che, Yu Yang, Hongchang Xie, Jing Tian

**Affiliations:** Department of Pediatrics, The Second Hospital of Jilin University, Changchun, China

**Keywords:** histone deacetylase 3, microRNA-296-5p, B-cell leukemia-XL, type 1 diabetes mellitus, peripheral blood mononuclear cells, apoptosis

## Abstract

Type 1 diabetes mellitus (T1DM) is a chronic autoimmune disease characterized by immune-mediated destruction of pancreatic beta-cells. Multiple microRNAs (miRNAs) have been implicated in T1DM pathogenesis. Although histone deacetylase 3 (HDAC3) has been reported to be involved in T1DM, the underlying mechanisms remain to be further elucidated. This study was designed to investigate the potential regulatory role of *Hdac3* on T1DM progression. The expression of *miR-296-5p* and B-cell leukemia-XL (BCL-XL) was determined using RT-qPCR and Western blot assay in peripheral blood mononuclear cells (PBMCs) of patients with T1DM, tumor necrosis factor-α (TNF-α)- and cycloheximide (CHX)-induced cell model, and streptozotocin (STZ)-induced rat model. The binding affinity between *miR-296-5p* and *Bcl-xl* was verified by using dual-luciferase reporter gene assay, and the binding between *Hdac3* and the promoter region of *miR-296-5p* was validated using chromatin immunoprecipitation assay. Western blot analysis and flow cytometry were conducted to assess the apoptotic events of lymphocytes. *miR-296-5p* expression was downregulated while BCL-XL expression was upregulated in PBMCs of patients with T1DM. An adverse correlation was identified between *miR-296-5p* and *Bcl-xl* in mouse TE15 B lymphocytes. *Bcl-xl* was further validated to be targeted and negatively regulated by *miR-296-5p* in 293 T cells. *Hdac3* inhibited *miR-296-5p* expression by binding to its promoter region. The effects of overexpressed *Hdac3* on lymphocyte apoptosis was counterweighed *via* downregulation of *Bcl-xl* or upregulation of *miR-296-5p*, the mechanism of which was further validated in a rat model of DM. Taken together, the *Hdac3*-mediated upregulation of *Bcl-xl via* inhibiting *miR-296-5p* promoter activity enhanced the anti-apoptotic capacity of lymphocytes to accelerate the occurrence of T1DM.

## Introduction

Type 1 diabetes mellitus (T1DM) is an autoimmune disorder characterized by immune-mediated destruction of pancreatic islet β-cells (Vatanen et al., 2018). The incidence of T1DM has drastically increased since 1950 worldwide (Craig et al., 2019). Patients with T1DM possess a higher risk to suffer from epilepsy compared with healthy individuals (Dafoulas et al., 2017). Additionally, coronary heart disease is the main factor responsible for the mortality of patients with T1DM that is closely related to insulin resistance (Bjornstad et al., 2018). Therefore, it is of great importance to develop novel and effective therapeutic strategies for T1DM treatment.

The occurrence and development of DM have been suggested to be closely correlated with histone deacetylases (HDACs; Zhang et al., 2019b). HDACs have been defined as a group of enzymes that play significant roles in mediating multiple processes at cellular and molecular levels (Merarchi et al., 2019). One of its members, *Hdac3*, has been reported to manage the gene expression in various tissues related to lipid metabolism (Davalos-Salas et al., 2019). In a mouse model of DM, the inhibition of *Hdac3* has been reported to activate NF-E2-related factor 2 to alleviate liver damage caused by DM (Zhang et al., 2018).

More importantly, *miR-296* has been revealed as one of the differentially expressed miRNAs in association with *Hdac3* according to microarray analysis in a previous study (Wang et al., 2016). Interestingly, *miR-296-5p* has been elucidated to be functional to diabetic wound healing, suggesting its potential as an effective molecular target in DM (Liu et al., 2019). As revealed from bioinformatics analysis prior to our study, B-cell leukemia-XL (*Bcl-xl*) was predicted to be targeted by *miR-296-5p*, suggesting a possible regulatory relation between *Bcl-xl* and *miR-296-5p*. *Bcl-xl*, localized in the mitochondria, belongs to the anti-apoptotic BCL-2 family, which has been reported to be implicated in regulating cell death and cellular functions (Li et al., 2020b). Apoptosis of β-cells has been recognized as a critical pathway underlying T1DM progression where the anti-apoptotic protein BCL-XL was identified as an important player in T1DM progression (Anuradha et al., 2014). The glucose signaling in pancreatic β-cells has been indicated to be restricted by *Bcl-xl* (Luciani et al., 2013). In addition, upregulation of *Bcl-xl* has been observed in peripheral blood mononuclear cells (PBMCs) in patients with T1DM (de Oliveira et al., 2012), but the upstream regulators of *Bcl-xl* remains uncharacterized. Gene expression alternation in PBMCs has also been recognized to provide a novel insight into identifying new biomarkers or treatment modalities for T1DM (Li et al., 2019). Therefore, in this study, we aimed to validate whether *Hdac3* could inhibit the apoptotic events of lymphocytes through *miR-296-5p* or *Bcl-xl* signaling pathway to increase the occurrence of T1DM.

## Materials and Methods

### Ethics Statement

All procedures concerning human samples were approved by the Ethics Committee of the Second Hospital of Jilin University. This study was performed based on the *Declaration of Helsinki* principles. The signed informed consents were obtained from all participants or their family members. All experimental procedures involving animals were approved by the Animal Care and Use Committee of the Second Hospital of Jilin University and in accordance with the *Guide for the Care and Use of Laboratory Animals* published by the National Institutes of Health.

### Study Subjects

PBMCs were obtained from patients with T1DM and age-matched healthy individuals as a control to analyze the expression of *miR-296-5p* and *Bcl-xl*. Thirty patients with T1DM (14 males and 16 females; average age 43.07 ± 5.56 years) who met the T1DM classification criteria revised by the American Diabetes Association in 1997 and 30 healthy volunteers (15 males and 15 females; average age 41.77 ± 8.35 years) were recruited in this study. Patients with ketoacidosis and delayed acidosis combined with nephropathy, proliferative retinopathy, diabetic foot syndrome, autonomic neuropathy, cardiovascular disease, and other diabetes complications were excluded from the study. For the controls, alcoholics, smokers, overweight and/or obese, who have a family history of diabetes, infection, hypertension, or long-term medication were also excluded from the study.

### Sample Collection and Isolation of PBMCs

An amount of 10 ml peripheral blood sample was collected from participants using EDTA-K2 tubes and processed within 2 h after collection. Ficoll-Hypaque (Sigma, St. Louis, MO, USA) was used to separate PBMCs by density-gradient method (Wang et al., 2018).

### Culture and Activation of Lymphocytes *in vitro*

PBMCs were cultured in Roswell Park Memorial Institute 1,640 medium containing 10% fetal bovine serum (FBS), 2 mmol-L l-glutamine, 100 U/ml penicillin, and 100 μg/ml streptomycin. Cells (10^6^ cells/ml) were cultured in a 24-well plate in an incubator containing 5% CO_2_ at 37°C for 3 days. Cell apoptosis was activated by the treatment with tumor necrosis factor-*α* (TNF-α) and cycloheximide (CHX; Zhang et al., 2019a; Li et al., 2020a).

### Cell Culture

TE15 cells were purchased from American Type Culture Collection (ATCC) and cultured in Dulbecco’s modified eagle medium (DMEM) with 20% FBS and 5% penicillin or streptomycin. The 293 T cells were purchased from ATCC and cultured in DMEM added with 10% FBS and 5% penicillin or streptomycin.

### RNA Isolation and Quantitation

Total RNA from tissues or cells was extracted using the miRNeasy Mini kit (QIAGEN, GmbH, Hilden, Germany) and subsequently quantified using NanoDrop ND-1000 Spectrophotometer (NanoDrop Products, Wilmington, DE, USA), whereas RNA integrity was evaluated by microfluidic electrophoresis. For reverse transcription (RT) of mRNA, 1 μg of RNA was synthesized into cDNA at 42°C for 50 min by using the TaqMan RT reagent (Roche, Canton of Basel, Switzerland). For the RT of miRNA, specific stem-loop primers were used to synthesize cDNA. The primers used in PCR were all synthesized by Sigma (Santa Clara, CA, USA; Table 1). Glyceraldehyde-3-phosphate dehydrogenase (*Gapdh*) was used as the internal reference primer for *Bcl-xl* and *Hdac3* genes, whereas *U6* was used for *miR-296-5p*. The relative transcription level of the target gene mRNA was calculated using the relative quantitative method (2^−△△CT^ method; Ayuk et al., 2016).

**Table 1:** Primer sequences used in RT-qPCR analysis.

Primer	Primer sequences (5'-3')	
*Gapdh*	F: TGATGGGTGTGAACCACGAG	R: TCAGTGTAGCCCAAGATGCC
*U6*	F: CTCGCTTCGGCAGCACA	R: AACGTTCACGAATTTGCGT
*Bcl-xl*	F: CTGAATCGGAGATGGAGACC	R: TGGGATGTCAGGTCACTGAA
*Hdac3*	F: CACCCTATGAAGCCCCATCG	R: GAGACCGTAATGCAGGACCAG
*miR-296-5p*	F: CGACGAGGGCCCCCCCT	R: GTATCCAGTGCAGG GTCCGA

RT-qPCR, reverse transcription-quantitative polymerase chain reaction; Gapdh, glyceraldehyde-3-phosphate dehydrogenase; *Bcl-xl*, B-cell leukemia-XL; *Hdac3*, histone deacetylase 3; *miR-296-5p*, microRNA-296-5p; F, forward; R, reverse.

### Western Blot Assay

PBMCs were added with 1 ml of cell lysis solution for the total protein isolation, and the protein concentration of each sample was measured using a bicinchoninic acid kit (20201ES76, Shanghai Yeasen Bio Technologies Co., Ltd., Shanghai, China). The sample was mixed with loading buffer and boiled at 100°C for 5 min. Following the ice bath and centrifugation, the protein was separated by lauryl sulfate-polyacrylamide gel electrophoresis and electro-transferred onto a nitrocellulose membrane. After it was blocked with 5% skimmed milk powder at 4°C overnight, the membrane was incubated with primary antibody rabbit anti-mouse BCL-XL (ab32370, 1: 2000), fasL (ab15285, 1: 2000), Bad (ab32445, 1: 2000), cleaved-caspase3 (C-caspase3; ab49822, 1: 500), Bcl-2 (ab59348, 1: 1000), and HDAC3 (ab32369, 1: 1000) at 4°C overnight. The membrane was washed thrice with tris-buffered saline tween, each time for 5 min. Horseradish peroxidase-labeled goat anti-rabbit immunoglobulin G (IgG; ab6721, 1: 5000) was then added and incubated with the membrane at room temperature for 1 h. Finally, the membrane was developed using enhanced chemiluminescence reagents. The above antibodies were purchased from Abcam Inc. (Cambridge, UK). The gray value of each band was analyzed by Quantity One software. GAPDH was used as an internal reference for the relative quantitative analysis of the target protein.

### Cell Transfection

PBMCs were seeded into a 6-well plate 24 h prior to transfection until cell reached about 70% confluence. Lipofectamine 2000 (11,668,019, Thermo Fisher Scientific, Waltham, MA, USA) was diluted to 20 μl in 500 μl serum-free medium and incubated for 5 min at room temperature. The plasmids and liposomes were gently mixed and incubated at room temperature for 20 min. Cells were washed three times with serum-free medium, followed by the addition of 2 ml of serum-free medium and then incubated with the above-mentioned sequence liposome mixture for 5–24 h. Next, 20% antibiotic-free DMEM was added and incubated for 48 h. The medium was renewed after 6 h of transfection and the cells were collected after 48 h of culture for subsequent experiments. The TE15 lymphocytes were transfected with *miR-296-5p* inhibitor, or the mimic and negative control (NC) plasmids. After that, the cells were transfected with *miR-296-5p* inhibitor, small interfering RNA (siRNA) targeting *Bcl-xl* (si*Bcl-xl*), and the corresponding NCs. Finally, the PBMCs were transfected with vectors pCMV-*hdac3* and si*Bcl-xl*, or the corresponding NC plasmids. The PBMCs were transfected with vectors and pCMV-*Bcl-xl* plasmids, respectively. Afterward, PBMCs were transfected with vector and pCMV-*Hdac3* and si*Hdac3* and siNC plasmids.

### Chromatin Immunoprecipitation Assay

The enrichment of *Hdac3* in the promoter region of *miR-296-5p* was determined using the ChIP kit (Millipore, Boston, MA, USA). Cells in the logarithmic growth phase were fixed using 1% formaldehyde for 10 min at room temperature to allow the DNA-protein crosslink formation. Next, cells were ultrasonicated and centrifuged at 13000 rpm at 4°C (some DNA fragments were retained as INPUT), and the supernatant was collected and aliquoted into three tubes, followed by the addition of IgG and target protein-specific antibody HDAC3 (ab32369, 1: 1000) and incubated at 4°C overnight. The above antibodies were purchased from Abcam Inc. (Cambridge, UK). Next, Protein Agarose or Sepharose was used to precipitate the endogenous DNA-protein complex, followed by centrifugation with the supernatant discarded. The non-specific complexes were washed off and the retained complex was allowed to stay overnight at 65°C for the de-crosslinking process. Subsequently, the DNA fragments were collected by phenol/extraction and purification, with INPUT as an internal reference. The specific primers in the promoter region of *miR-296-5p* (Table 1) were used to examine the binding between *Hdac3* and the promoter region of *miR-296-5p*.

### Dual-Luciferase Reporter Gene Assay

Wild type (WT) of *miR-296-5p* 3'-untranslated region (3'-UTR) was synthesized and the WT target site was mutated. The synthetic target gene fragments WT and mutant (MUT) were subsequently inserted into the pmiR-RB-REPORT™ vector (Guangzhou RiboBio Co., Ltd., Guangzhou, China). At the same time, empty plasmids were transfected as control and the correct luciferin enzyme reporter plasmids WT and MUT were co-transformed with NC mimic, *miR-296-5p* mimic, inhibitor NC, or *miR-296-5p* inhibitor into HEK293T cells. Thereafter, the cells were collected and lysed after 48 h of transfection, followed by centrifugation for 3–5 min with the supernatant collected. The relative light unit value was measured using Renilla luciferase detection kit (YDJ2714, Shanghai Yuduo Biotechnology Co., Ltd., Shanghai, China) with firefly luciferase as the internal reference, followed by analysis using dual-luciferase reporting analysis system (Promega Co., Madison, WI, USA).

### Flow Cytometry

After cell transfection for 48 h, cells were detached using 0.25% trypsin and centrifuged. Next, cells were resuspended using annexin-V/propidium iodide (PI) staining solution using annexin-V-fluorescein-5-isothiocyanate kit (556547, Shanghai Solja Technology Co., Ltd., Shanghai, China) and incubated for 15 min. Next, cells were gently mixed and added with 15 ml 4-(2-hydroxyethyl)piperazine-1-ethanesulfonic acid buffer. The apoptotic events were evaluated using flow cytometer (Bio-Rad ZE5, Bio-Rad Laboratories, Hercules, CA, USA) at a maximum absorption wavelength of 488 nm and excitation wavelength of 525 nm. The maximum absorption and emission wavelengths of the PI-DNA were 535 and 615 nm, respectively. For apoptosis induction, apoptosis inducers (20 ng/μl TNF-α and 10 μg/μl CHX; 1,000: 1) were added to the culture medium 24 h after transfection, and the cells were collected and subjected to flow cytometry for apoptosis detection.

### Animal Model Development

Male Sprague Dawley rats (8 weeks, Experimental Animal Center of the Second Hospital of Jilin University) were acclimated for 7 days with free access to food and drinking water in a clean polypropylene cage at (21 ± 2)°C under relative humidity of (50 ± 5)% in a room exposed to artificial 12/12-h light-dark cycles. Eight rats were injected with solvent (62 mg/kg) serving as the sham group. Then, intraperitoneal injection of streptozocin (STZ; 62 mg/kg; Sigma) in citric acid (0.01 M, pH = 4.5) was performed on another 16 rats to induce DM. Thereinto, eight DM rats were injected with lentivirus harboring sh-*Hdac3 via* tail vein on a daily basis. On the first after inducement, mice were given free access to water containing 5% glucose to avoid hypoglycemia. The glucose and insulin levels of rats were measured after 8 weeks. PBMCs were then isolated from serum of rats for following experiments.

### Statistical Analysis

SPSS version 21.0 (IBM Corp., Armonk, NY, USA) was used for statistical analysis. The measurement data were expressed as mean ± standard deviation. Comparison between the two groups complying with the normal distribution and homogeneity of variance was tested using the unpaired *t*-test. Data comparisons among multiple groups were analyzed using one-way ANOVA, followed by *post hoc* analysis (Turkey test and Bonferroni correction) to evaluate the statistical significance. Kruskal-Wallis (non-parametric) test was used for data with skewed distribution and then Wilcoxon test with Bonferroni correction was used to compare the mean value in the individual group. A value of *p* < 0.05 was considered statistically significant.

## Results

### *miR-296-5p* Was Downregulated and *Bcl-xl* Was Upregulated in PBMCs of Patients With T1DM

**Figure 1:**
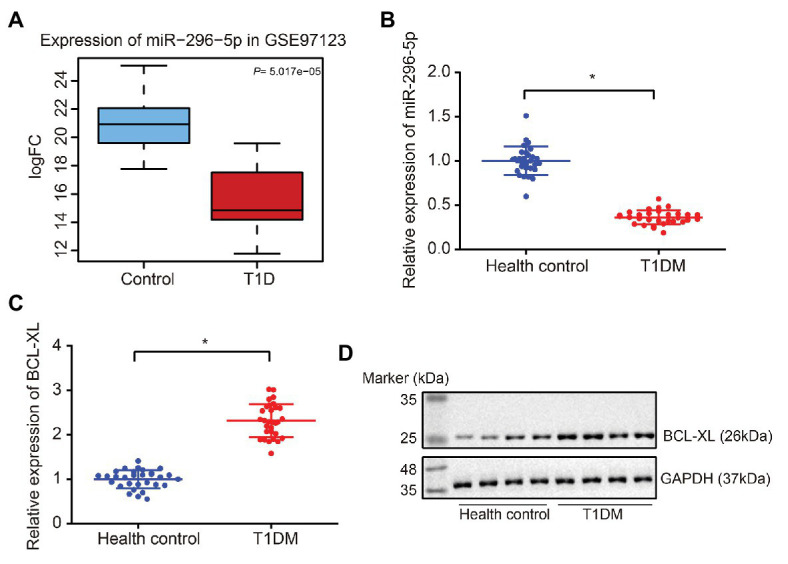
Downregulation of *miR-296-5p* and upregulation of BCL-XL in peripheral blood mononuclear cells (PBMCs) of patients with Type 1 diabetes mellitus (T1DM) are observed. **(A)** Box plot of *miR-296-5p* expression in normal samples (blue box on the left) and in T1DM samples (right box on the right) from microarray dataset GSE97123. **(B)** The expression of *miR-296-5p* in PBMCs of patients with T1DM and healthy individuals determined by RT-qPCR. U6 was used as an internal reference. **(C)** The expression of *Bcl-xl* in PBMCs of patients with T1DM and healthy individuals determined by RT-qPCR. *Gapdh* was used as an internal reference. **(D)** The protein expression of BCL-XL in PBMCs of patients with T1DM and healthy individuals was determined by Western blot assay. GAPDH was used as an internal reference. ^*^p < 0.05 compared with the control. The results were measurement data and expressed as mean ± standard deviation, and data comparison between two groups was performed using unpaired sample *t*-test, *n* = 30.

Initially, a box plot was drawn on the *miR-296-5p* expression in microarray dataset GSE97123 from GEO database, followed by significant difference analysis using *t*-test. Results revealed significantly low expression of *miR-296-5p* in T1DM (Figure 1A). Subsequently, PBMCs from patients with T1DM and healthy individuals were isolated, and the expression of *miR-296-5p* and *Bcl-xl* in the samples was determined using reverse transcription quantitative polymerase chain reaction (RT-qPCR). The results showed that *miR-296-5p* was downregulated and *Bcl-xl* was upregulated in PBMCs of patients with T1DM compared to healthy individuals (Figures 1B,C). Results from Western blot analysis showed that the protein expression of BCL-XL was significantly increased in PBMCs of patients with T1DM compared to healthy individuals (Figure 1D), suggesting the low expression of *miR-296-5p* and high expression of *Bcl-xl* in PBMCs of patients with T1DM.

### *miR-296-5p* Targeted and Inhibited the Expression of *Bcl-xl*

**Figure 2:**
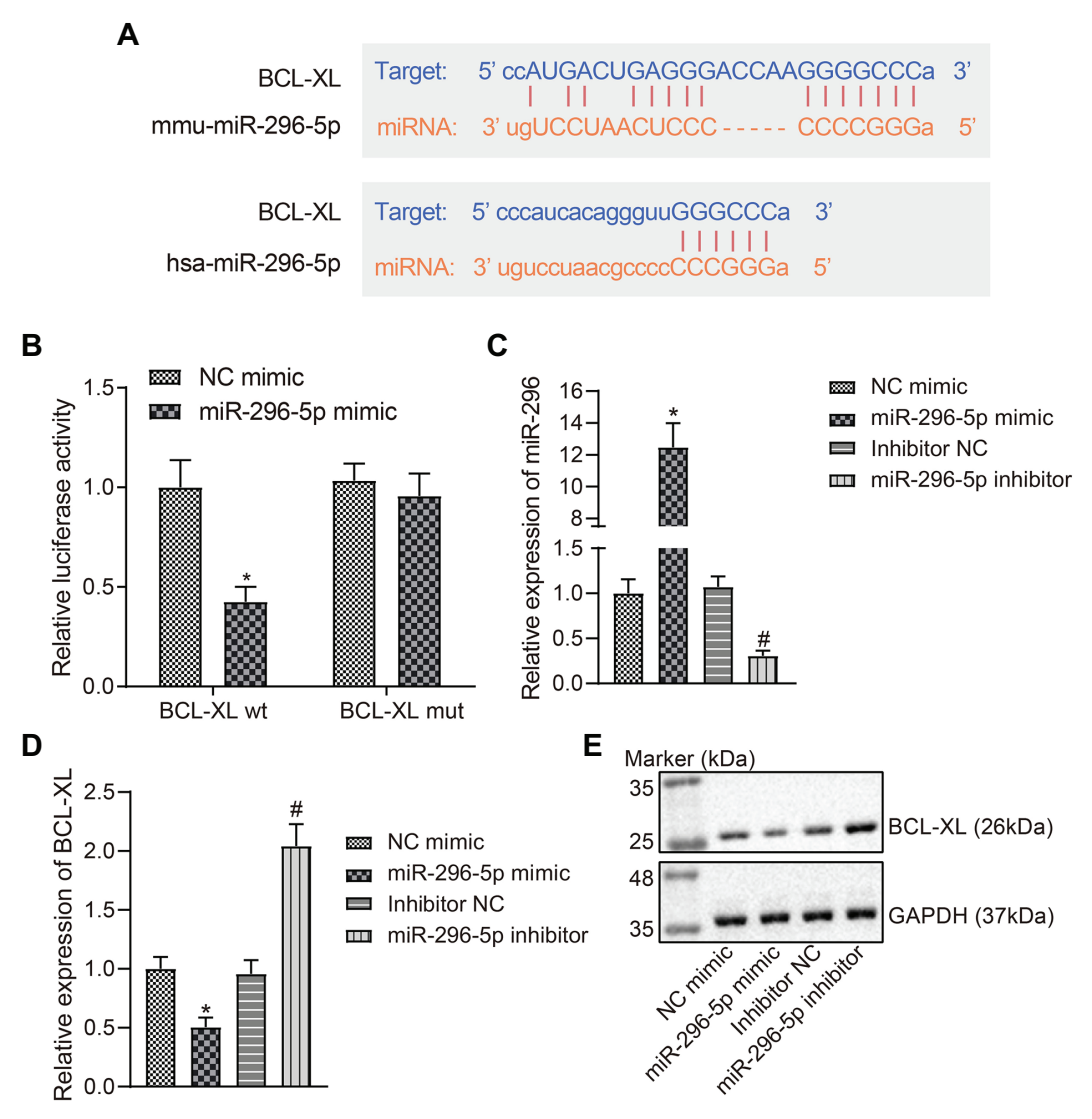
*miR-296-5p* directly targets *Bcl-xl*. **(A)** The binding site of hsa-*miR-296-5p* and *Bcl-xl* 3'-UTR predicted by Starbase software. **(B)** The luciferase activity of *Bcl-xl* in 293 T cells transfected with *miR-296-5p* mimic or NC mimic. **(C)** The expression of *miR-296-5p* in lymphocytes determined by RT-qPCR. **(D)** The mRNA expression of *Bcl-xl* in lymphocyte series determined by RT-qPCR. **(E)** The protein expression of BCL-XL normalized to GAPDH in lymphocyte series determined by Western blot assay. ^*^p < 0.05 compared with cells transfected with NC mimic, ^#^*p* < 0.05 compared with cells transfected with inhibitor NC. The results were measurement data and expressed as mean ± standard deviation, and data comparison between two groups was performed using an unpaired sample *t*-test. The cell experiment was repeated three times independently.

Based on the bioinformatics data obtained from the Starbase website, we predicted that *miR-296-5p* may target the 3'-UTR of *Bcl-xl* in both humans and mice (Figure 2A). Therefore, we constructed luciferase reporter vectors and corresponding MUT vectors for the 3'-UTR of *Bcl-xl* (Figure 2B). The results showed that *miR-296-5p* directly targeted 3'-UTR of *Bcl-xl* and diminished the expression of *Bcl-xl*.

In order to further investigate whether the expression of *miR-296-5p* and *Bcl-xl* showed a negative correlation *in vitro*, TE15 mouse B lymphocytic cell line was selected and the change regarding expression of *miR-296-5p* and BCL-XL was determined using RT-qPCR and Western blot assay after B lymphocytic series were transfected with NC mimic and *miR-296-5p* mimic (Figures 2C–E). The results showed that the expression of *miR-296-5p* and BCL-XL *in vitro* was significantly correlated in a negative manner, which was consistent with the results from PBMC samples. The expression of *miR-296-5p* in PBMCs was inhibited using *miR-296-5p* inhibitor, and the results showed that BCL-XL was upregulated at mRNA and protein levels (Figures 2C–E). To conclude, these results highlighted the inverse relation between *miR-296-5p* and *Bcl-xl*.

### *miR-296-5p* Promoted Lymphocyte Apoptosis by Targeting *Bcl-xl*

**Figure 3:**
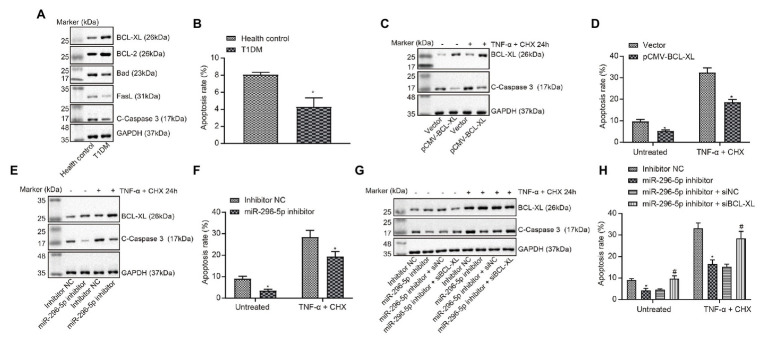
*miR-296-5p* overexpression enhances lymphocyte apoptosis by targeting *Bcl-xl*. **(A)** The expression of pro-apoptotic and anti-apoptotic proteins normalized to GAPDH in PBMCs of patients with T1DM determined by Western blot assay (*n* = 30). **(B)** Quantitative analysis of apoptosis of PBMCs in patients with T1DM compared with healthy individuals analyzed using flow cytometry. **(C)** The protein expression of BCL-XL and C-caspase3 normalized to GAPDH determined by Western blot assay after lymphocytes transfected with vector and pCMV-*Bcl-xl*. **(D)** Quantitative analysis of apoptosis of lymphocytes transfected with vector and pCMV-*Bcl-xl* analyzed using flow cytometry. **(E)** The protein expression of BCL-XL and C-caspase3 normalized to GAPDH determined by Western blot assay after lymphocytes transfected with *miR-296-5p* inhibitor or inhibitor NC. **(F)** Quantitative analysis of apoptosis of lymphocytes after transfected with *miR-296-5p* inhibitor or inhibitor NC evaluated by flow cytometry. **(G)** Rescue experiments on the protein expression of BCL-XL and C-caspase3 normalized to GAPDH determined by Western blot assay. **(H)** Rescue experiments on the apoptosis of lymphocytes evaluated by flow cytometry. ^*^p < 0.05 compared with cells transfected with control, vector, or inhibitor NC, ^#^*p* < 0.05 compared with cells transfected with *miR-296* inhibitor + si-NC. The results were measurement data and expressed as mean ± standard deviation. Data comparison between two groups was performed using unpaired sample *t*-test, and data comparisons among multiple groups were performed using one-way analysis of variance. The cell experiment was repeated three times independently.

The expression of proteins related to apoptosis in PBMCs of patients with T1DM was tested using Western blot analysis (Figure 3A). The results showed that the expression of pro-apoptosis proteins Bad and fasL was significantly decreased, and the expression of anti-apoptosis proteins BCL-XL and BCL-2 was significantly increased in PBMCs of patients with T1DM. The apoptosis of PBMCs in patients with T1DM was evaluated by flow cytometry (Figure 3B), and the results showed that the percentage of apoptotic cells was remarkably decreased in PBMCs of patients with T1DM compared with healthy individuals.

Next, the effects of *Bcl-xl* on apoptotic-related proteins and apoptosis levels in lymphocytes were evaluated (Figures 3C,D), and the results showed that the upregulation of *Bcl-xl* could inhibit apoptosis induced by TNF-α and CHX. Subsequently, the effects of *miR-296-5p* on apoptotic-related proteins and apoptosis levels in lymphocytes were determined using Western blot and flow cytometry (Figures 3E,F), and effect of downregulated *miR-296-5p* on lymphocyte apoptosis was consistent with the effect of upregulated *Bcl-xl*. Therefore, we speculated that *miR-296-5p* could target and diminish the expression of *Bcl-xl* to regulate lymphocyte apoptosis. To verify this hypothesis, we performed apoptosis-related rescue experiments (Figures 3G,H), and *Bcl-xl* was able to rescue the *miR-296-5p*-induced apoptosis. Taken together, these results suggested that *miR-296-5p* could promote lymphocyte apoptosis by targeting *Bcl-xl*.

### *Hdac3* Downregulated *miR-296-5p* by Binding to Its Promoter Region

**Figure 4:**
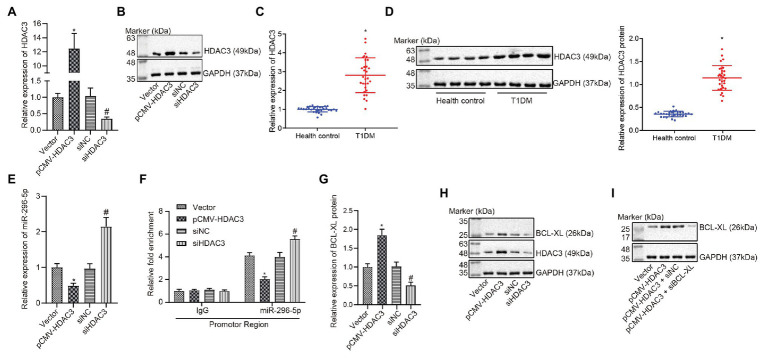
HDAC3 restricts the expression of *miR-296-5p* by binding to with its promoter region. **(A)** The expression of *Hdac3* determined by RT-qPCR. **(B)** The expression of HDAC3 determined by Western blot assay. **(C)** The expression of *Hdac3* in PBMCs of T1DM patients by RT-qPCR. **(D)** The expression of HDAC3 in PBMCs of T1DM patients by Western blot assay. **(E)** The expression of *miR-296-5p* determined by RT-qPCR when HDAC3 was upregulated and downregulated, respectively. **(F)** Inhibition of *miR-296-5p* promoter activity by *Hdac3* evaluated using ChIP-qPCR assay. **(G)** The expression of *Bcl-xl* determined by RT-qPCR when *Hdac3* was upregulated or downregulated, respectively. **(H)** The expression of BCL-XL determined by Western blot assay when HDAC3 was upregulated or downregulated, respectively. **(I)** Rescue experiments showed that HDAC3 upregulated the expression of BCL-XL by targeting *miR-296-5p*. ^*^p < 0.05 compared with cells transfected with vector, ^#^*p* < 0.05 compared with cells transfected with si-NC. The results were measurement data and expressed as mean ± standard deviation. Data comparison between two groups was performed using unpaired sample *t*-test, and data comparisons among multiple groups were performed using one-way analysis of variance. The cell experiment was repeated three times independently.

It has been reported that dysfunctional histone and transfer factors acetylation is correlated with pathogenesis of diabetes (Gray and De Meyts, 2005). *HDAC*s, including 18 genes, can mediate acetylation of histone (Blixt et al., 2017). To our knowledge, there are seven studies reporting the correlation between *Hdac3* and T1DM, ranking the first among the 18 genes. Meanwhile, *Hdac3* contributes to the occurrence of T1DM (Meier and Wagner, 2014). Results from the gene expression dataset have revealed that *Hdac3* is negatively correlated with *miR-296-5p* and can inhibit the occurrence of diabetes (Wang et al., 2016; Liu et al., 2019). Therefore, we inferred that *Hdac3* might be the most relevant *Hdac* with T1DM and there may be an interaction between *Hdac3* and *miR-296-5p*. Then, *Hdac3* was overexpressed or knocked down, followed by quantification of resultant expression patterns. Results showed that HDAC3 expression was elevated in response to pCMV- *Hdac3* yet diminished in response to si*Hdac3* (Figures 4A,B). Here, we found that the expression of HDAC3 was significantly increased in PBMCs of patients with T1DM than corresponding controls (Figures 4C,D). For verification purpose, the expression of *miR-296-5p* was evaluated when *Hdac3* was upregulated and downregulated, respectively. The results indicated that there was a significant negative correlation between *Hdac3* and *miR-296-5p* expression (Figure 4E).

In order to further investigate the effect of HDAC3 on the promoter activity of *miR-296-5p*, the ChIP-qPCR experiment was performed and the results showed that the promoter activity of *miR-296-5p* was significantly deteriorated when *Hdac3* was upregulated (Figure 4F). Based on the aforementioned study of *miR-296-5p* and *Bcl-xl*, the relationship between *Hdac3* and *Bcl-xl* was further explored, and the results showed that the expression of *Hdac3* was positively correlated with the expression of *Bcl-xl* (Figures 4G,H). The results also showed that *Hdac3* could regulate the expression of *Bcl-xl* by targeting *miR-296-5p* (Figure 4I). These results indicated that *Hdac3* could inhibit the expression of *miR-296-5p* by binding to its promoter region to reinforce the expression of *Bcl-xl*.

### HDAC3 Regulated BCL-XL by Mediating *miR-296-5p* to Suppress Apoptosis of Lymphocytes

**Figure 5:**
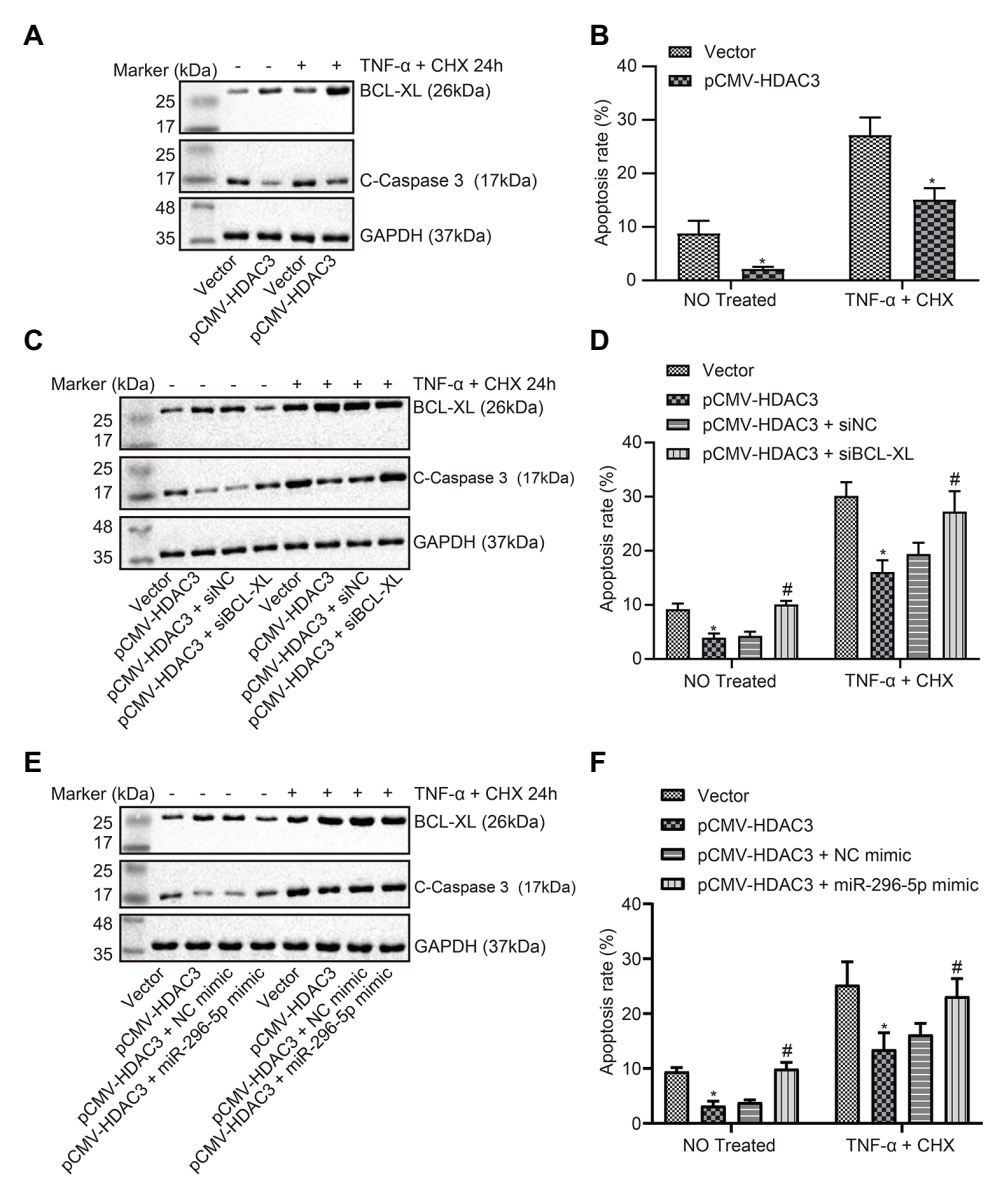
HDAC3-mediated *miR-296-5p* attenuates the apoptosis of lymphocytes *via* the regulation of BCL-XL. **(A)** The expression of BCL-XL and C-caspase3 normalized to GAPDH determined by Western blot assay when HDAC3 was upregulated in lymphocytes. **(B)** The effect on apoptosis evaluated by flow cytometry when *Hdac3* was upregulated in lymphocytes. **(C)** The effect of BCL-XL on the expression of C-caspase3 normalized to GAPDH after HDAC3 was downregulated in lymphocytes determined by Western blot assay. **(D)** The effect of *Bcl-xl* on apoptosis after *Hdac3* was upregulated in lymphocytes evaluated by flow cytometry. **(E)** The effect of *miR-296-5p* on the expression of C-caspase3 normalized to GAPDH after HDAC3 was upregulated in lymphocytes tested by Western blot assay. **(F)** The effect of *miR-296-5p* on apoptosis after *Hdac3* was upregulated in lymphocytes tested by flow cytometry. ^*^p < 0.05 compared with cells transfected with vector, ^#^*p* < 0.05 compared with cells transfected with pCMV-*Hdac3* + si-NC or pCMV-*Hdac3* + NC mimic. The results were measurement data and expressed as mean ± standard deviation. Data comparison between two groups was performed using unpaired sample *t*-test, and data comparisons among multiple groups were performed using one-way analysis of variance. The cell experiment was repeated three times independently.

The expression of apoptosis-related proteins was determined when HDAC3 was upregulated (Figure 5A), and the effect on apoptosis was evaluated by flow cytometry. The results showed that the effect of upregulated HDAC3 on apoptosis was consistent with the effect of downregulated *miR-296-5p* on apoptosis (Figure 5B). Results also showed that BCL-XL expression was restored when HDAC3 was upregulated and BCL-XL downregulated at the same time (Figure 5C), and the results from flow cytometry showed that the protein levels of apoptosis in the presence of both upregulated HDAC3 and downregulated BCL-XL were restored (Figure 5D). At the same time, the expression of BCL-XL was restored when both HDAC3 and *miR-296-5p* were upregulated (Figure 5E), and protein levels of apoptosis in response to upregulated HDAC3 and downregulated BCL-XL were restored (Figure 5F). These results consistently showed that HDAC3 could regulate BCL-XL by mediating the expression of *miR-296-5p* to inhibit lymphocyte apoptosis.

### *Hdac3* Knockdown Curbed the Apoptosis of PBMCs in Rats With DM

**Figure 6:**
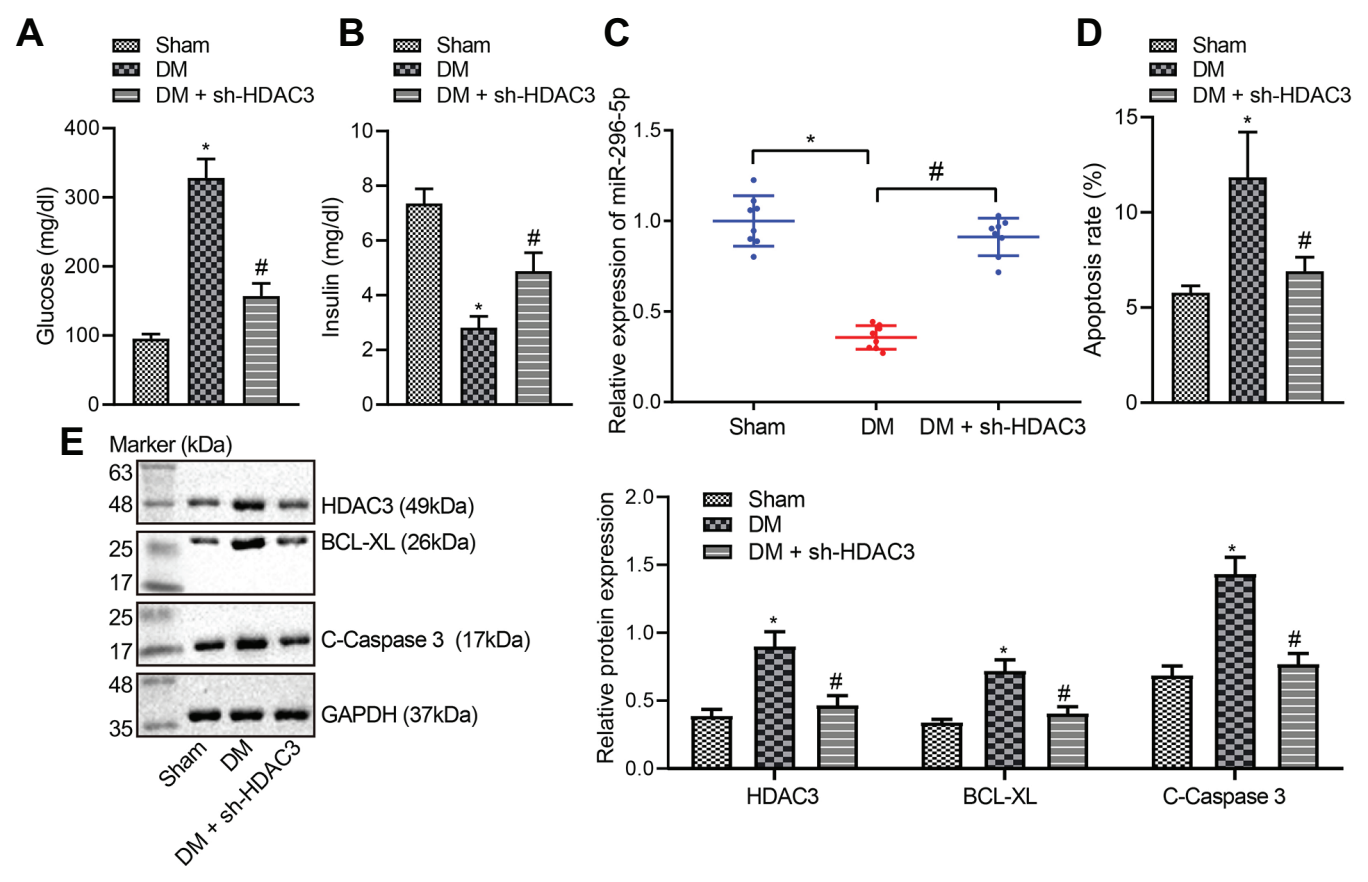
shRNA-mediated silencing of HDAC3 alleviates DM-induced PBMC apoptosis *in vivo*. **(A)** Glucose level of rats. **(B)** Insulin levels of rats. **(C)** *miR-296-5p* expression in PBMCs of rats determined by RT-qPCR. **(D)** Quantitative analysis of PBMC apoptosis identified by flow cytometric analysis. **(E)** The expression of HDAC3, BCL-XL, and C-caspase-3 in PBMCs normalized to GAPDH determined by Western blot analysis. ^*^p < 0.05 compared with sham-operated rats, ^#^*p* < 0.05 compared with rats with DM. Data comparisons among multiple groups were performed using one-way analysis of variance. *n* = 8.

Glucose and insulin levels of rats in each group were measured (Figures 6A,B) and *miR-296-5p* expression in PBMCs of rats was determined by RT-qPCR (Figure 6C). Results revealed significantly higher glucose level, lower insulin level, and diminished *miR-296-5p* expression in rats with DM while further addition of sh-*Hdac3* reversed the results. PBMC apoptosis was then evaluated by flow cytometric analysis (Figure 6D) and Western blot analysis (Figure 6E). It was found that PBMC apoptosis was potentiated in rats with DM accompanied by upregulation of HDAC3, BCL-XL, and C-caspase-3, all of which were reversed by delivery of sh-*Hdac3*. Taken together, shRNA-mediated silencing of HDAC3 significantly alleviated DM-induced PBMC apoptosis in rats.

## Discussion

DM has been revealed to be closely related to HDACs. *Hdac3* inhibitors have been proposed as a potential therapeutic candidate in managing DM (Meier and Wagner, 2014; Khan and Jena, 2015). The present study was designed to explore the effect of *Hdac3* on the expression of *miR-296-5p* and *Bcl-xl*, as well as to identify its potential role in T1DM. This study provided evidence demonstrating that *Hdac3* could inhibit the apoptosis of lymphocytes by restricting *miR-296-5p* to upregulate *Bcl-xl*, thereby promoting the occurrence of T1DM.

Initially, our results demonstrated that *miR-296-5p* was downregulated and BCL-XL was upregulated in PBMCs of patients with T1DM. The regulation of gene expression in PBMCs has been widely indicated to offer new and better treatment modalities for patients with T1DM (Li et al., 2019). Differentially expressed miRNAs in PBMCs have been deciphered to function as promising biomarkers of T1DM, providing new insights into the understanding and the underlying molecular mechanism (Takahashi et al., 2014). Consistently, *miR-296-5p* expression has been reported to show a significant reduction in DM tissues in comparison to corresponding normal tissues, while healing of diabetic wounds has been shown to be facilitated by the overexpression of *miR-296-5p* by targeting sodium-glucose transporter 2 (Liu et al., 2019). A similar expression profile of multiple miRNAs has also been identified in patients suffering from T1DM, including *miR-150*, *miR-146a*, and *miR-424*, with great clinical application potential (Wang et al., 2018). In addition, a previous report has documented that *Bcl-xl* is highly expressed in PBMCs of patients with T1DM, which leads to the development of T1DM (de Oliveira et al., 2012). Moreover, according to experimental data of the present study, it was verified that *miR-296-5p* could target *Bcl-xl* and negatively regulate the expression of *Bcl-xl*, which resulted in enhanced apoptosis of lymphocytes. B lymphocytes have been suggested to participate in β-cell destruction in patients with T1DM (Deng et al., 2016). Notably, targeting dysfunctional β-cell signaling has been proposed as a novel target for the management of T1DM (Fenske and Kimple, 2018). Additionally, lymphocytes have also been demonstrated to be increased in patients with T1DM in association with poor glycemic control related to the occurrence of cardiovascular disease and coronary syndromes (Giubilato et al., 2011), supporting the validation of our findings.

Subsequently, diminished levels of pro-apoptotic proteins, Bad and fasL, were detected in PBMCs collected from patients with T1DM accompanied by elevated expression of anti-apoptotic proteins, BCL-XL and BCL-2. In agreement with our results, previous work has reported that the upregulation of Bad and fasL along with downregulation of BCL-XL has been detected in PBMCs of patients with T1DM, serving as potential biomarkers of clinical remission (de Oliveira et al., 2012). Furthermore, vector and pCMV-*Bcl-xl* were delivered into lymphocytes to unravel the involvement of *Hdac3* in T1DM. It was found that *Hdac3* downregulated the expression of *miR-296-5p* by binding to the promoter region of *miR-296-5p*. Many studies showed that as HDACs function as part of multi-protein complexes that deacetylate histone tails and modify chromatin structure and gene repression, HDAC inhibitors decreased miRNA promoter methylation (Mazzu et al., 2019), and we thus speculated that *Hdac3* also affects the acetylation level of the promoter region of *miR-296-5p* and to reduce its expression. Additionally, HDAC3 exerted an inhibitory effect on lymphocyte apoptosis by upregulating BCL-XL expression *via miR-296-5p*. Meanwhile, downregulation of BCL-XL or overexpression of *miR-296-5p* was shown to override the effects of overexpressed HDAC on protein expression of apoptosis-related factors and flow cytometric analysis of lymphocyte apoptosis. HDAC enzyme plays a regulatory role in chromatin structure and metabolic enzyme acetylation in mitochondria and cytosol, shedding light on the intrinsic role of HDAC inhibitors as therapeutic strategies for DM (Christensen et al., 2011; Makkar et al., 2019). Class I HDAC inhibition has been reported to increase insulin secretion and prevent pancreatic beta cell from apoptosis (Peng et al., 2019). Concordantly, the downregulation of *Hdac3* has been considered as a strategy for developing new ways for the treatment of diabetes (Meier and Wagner, 2014). Previous data have shown that inhibition of *Hdac3* prevents cytokine-induced beta-cell apoptosis, which is important to the etiology of T1DM (Chou et al., 2012). In addition, *Hdac3* can integrate microbiota-derived signals to control intestinal homeostasis in intestinal epithelial cells (IECs), where disruption of *Hdac3* causes weight loss and improves metabolic profile, suppressing the diet-induced obesity (Whitt et al., 2018). Likewise, inhibition of HDACs has been highlighted to improve myocardial function in a model of diabetic mice by increasing GLUT1 acetylation and p38 phosphorylation (Chen et al., 2015). Moreover, suppression of *Hdac3* has been elucidated to prevent diabetic cardiomyopathy in mouse models through epigenetic regulation of the DUSP5-ERK1/2 pathway (Xu et al., 2017). Therefore, it could be concluded from our collective experimental data both *in vitro* and *in vivo*, as well as the previous reports, that HDAC3 could harbor the potential to impede *miR-296-5p* expression and lead to the aggravation of T1DM.

## Conclusion

**Figure 7:**
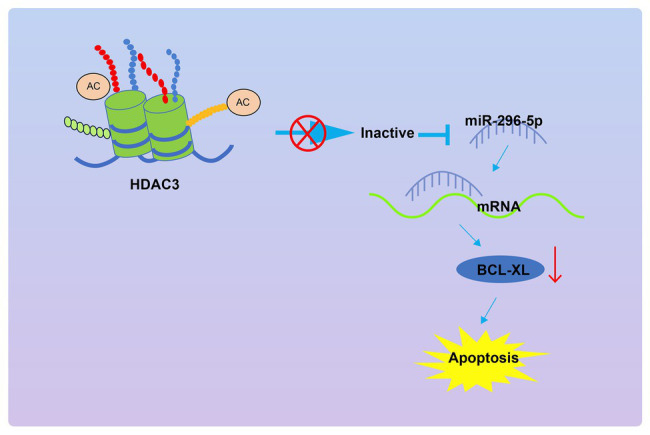
The graphical summary of the function and mechanism of *Hdac3* on the apoptosis of PBMCs in T1DM *via* the *miR-296-5p/Bcl-xl* axis.

In conclusion, the present study demonstrates that *Hdac3* could upregulate *Bcl-xl* by repressing *miR-296-5p* expression, leading to inhibited apoptosis of lymphocytes and subsequently promoted occurrence of T1DM (Figure 7). This study provides new insights into mechanisms underlying T1DM and novel potential therapeutic targets for patients with T1DM. Despite these promising results, the molecular mechanisms underlying apoptosis of PBMCs in the context of T1DM are still not well-characterized. Therefore, more in-depth investigations are required to reveal the molecular mechanisms underlying T1DM development, as a pre-requisite for better application in a clinical setting in the future.

## Data Availability Statement

The raw data supporting the conclusions of this article will be made available by the authors, without undue reservation, to any qualified researcher.

## Ethics Statement

All procedures concerning human samples were approved by the ethics committee of the Second Hospital of Jilin University. The written informed consents were obtained from all participants or their family members.

## Author Contributions

QH and GC designed the study. YY and HX collated the data, carried out data analyses, and produced the initial draft of the manuscript. QH and JT contributed to drafting the manuscript. All authors contributed to the article and approved the submitted version.

### Conflict of Interest

The authors declare that the research was conducted in the absence of any commercial or financial relationships that could be construed as a potential conflict of interest.
