# Corrigendum: Histone Deacetylase 3 Aggravates Type 1 Diabetes Mellitus by Inhibiting Lymphocyte Apoptosis Through the *microRNA-296-5p/Bcl-xl* Axis

**DOI:** 10.3389/fgene.2021.715061

**Published:** 2021-09-22

**Authors:** Qibo Hu, Guanghua Che, Yu Yang, Hongchang Xie, Jing Tian

**Affiliations:** Department of Pediatrics, The Second Hospital of Jilin University, Changchun, China

**Keywords:** histone deacetylase 3, microRNA-296-5p, B-cell leukemia-XL, type 1 diabetes mellitus, peripheral blood mononuclear cells, apoptosis

In the original article, there was a mistake in ***Table 1*** as published. We misplaced the forward primer and reverse primer when typesetting, which has been adjusted. The corrected ***Table 1*** appears below.

**Table 1:** Primer sequences used in RT-qPCR analysis.

**Primer**	**Primer sequences (5′-3′)**	
*Gapdh*	F: TGATGGGTGTGAACCACGAG	R: TCAGTGTAGCCCAAGATGCC
*U6*	F: CTCGCTTCGGCAGCACA	R: AACGTTCACGAATTTGCGT
*Bcl-xl*	F: CTGAATCGGAGATGGAGACC	R: TGGGATGTCAGGTCACTGAA
*Hdac3*	F: CACCCTATGAAGCCCCATCG	R: GAGACCGTAATGCAGGACCAG
*miR-296-5p*	F: CGACGAGGGCCCCCCCT	R: GTATCCAGTGCAGG GTCCGA

*RT-qPCR, reverse transcription-quantitative polymerase chain reaction; Gapdh, glyceraldehyde-3-phosphate dehydrogenase; Bcl-xl, B-cell leukemia-XL; Hdac3, histone deacetylase 3; miR-296-5p, microRNA-296-5p; F, forward; R, reverse*.

Additionally, there was also a mistake in the text of the published article. We are sorry that the previous method description was not clear enough. Now we have modified the related method description: “For reverse transcription (RT) of mRNA, 1 μg of RNA was synthesized into cDNA at 42°C for 50 min by using the TaqMan RT reagent (Roche, Canton of Basel, Switzerland). For the RT of miRNA, specific stem-loop primers were used to synthesize cDNA.”

A correction has been made to ***Methods***, ***RNA Isolation and Quantitation***, ***Paragraph 1***:

Total RNA from tissues or cells was extracted using the miRNeasy Mini kit (QIAGEN, GmbH, Hilden, Germany) and subsequently quantified using NanoDrop ND-1000 Spectrophotometer (NanoDrop Products, Wilmington, DE, USA), whereas RNA integrity was evaluated by microfluidic electrophoresis. For reverse transcription (RT) of mRNA, 1 μg of RNA was synthesized into cDNA at 42°C for 50 min by using the TaqMan RT reagent (Roche, Canton of Basel, Switzerland). For the RT of miRNA, specific stem-loop primers were used to synthesize cDNA.

The authors apologize for these errors and state that this does not change the scientific conclusions of the article in any way. The original article has been updated.

## Publisher's Note

All claims expressed in this article are solely those of the authors and do not necessarily represent those of their affiliated organizations, or those of the publisher, the editors and the reviewers. Any product that may be evaluated in this article, or claim that may be made by its manufacturer, is not guaranteed or endorsed by the publisher.

